# Macroscopic and ultrasonographic anatomy of the brachial plexus in the American kestrel (*Falco sparverius*)

**DOI:** 10.1007/s11259-026-11506-8

**Published:** 2026-09-19

**Authors:** Ana Paula Morel, Maria Lúcia Rösler, Ana Júlia Fazenda de Souza, Luana Célia Stunitz da Silva, Raqueli Teresinha França

**Affiliations:** 1 Universidade Federal de Pelotas, Faculdade de Veterinária, Campus Universitário Capão do Leão, RS Capão do Leão, 96160-000 Brazil; 2 Departamento de Biociências, Universidade Federal do Paraná – Setor Palotina, Palotina, Paraná Brazil; 3 Departamento de Clínicas Veterinárias, Universidade Federal de Pelotas, Campus Universitário Capão do Leão, Capão do Leão, RS Brazil

**Keywords:** raptors, innervation, regional anesthesia, ultrasonography

## Abstract

The American kestrel (*Falco sparverius*) is a small raptor frequently admitted to wildlife rehabilitation centers, often due to traumatic injuries requiring surgical intervention. Regional anesthesia techniques, particularly brachial plexus blocks, may improve perioperative management; however, species-specific anatomical and ultrasonographic data remain limited. This study aimed to characterize the macroscopic and ultrasonographic anatomy of the brachial plexus in *F. sparverius* and to assess the distribution of injectate following ultrasound-guided administration. Nineteen kestrels were allocated into three groups: anatomical dissection (n = 6), ultrasonographic evaluation (n = 8), and cadaveric ultrasound-guided dye injection 0.5 mL kg⁻¹ of a lidocaine–methylene blue solution resulted in consistent dye distribution within the plexus location (n = 5). The brachial plexus was formed by the ventral rami of spinal nerves C13–T2, organized into dorsal and ventral fascicles. Ultrasonographically, the brachial plexus appeared as small hypoechoic structures beneath the superficial and deep pectoral muscles using a ventral approach. These findings demonstrate that the brachial plexus of *F. sparverius* follows a typical avian pattern with species-specific variations and that the ventral ultrasonographic approach allows reliable visualization and accurate injectate delivery. This study provides anatomical and imaging data to support the development of regional anesthesia techniques in small raptors.

## Introduction

The American kestrel (*Falco sparverius*) is a small falcon (80–160 g) distributed from Alaska to southern South America, inhabiting diverse environments, including urban areas. It is an opportunistic predator feeding on arthropods, small vertebrates, and amphibians (Ferguson-Lees and Christie, 2001).

This species is one of the most frequently admitted raptors to rehabilitation centers in the Americas, often due to trauma, particularly wing fractures requiring surgical intervention (Morishita et al., 1998; Morel et al., 2025). Knowledge of the brachial plexus is essential for safe anesthesia and surgery, and regional anesthesia has become increasingly important for analgesic management in veterinary medicine (Akbulut et al., 2017; Hawkins and Paul-Murphy, 2011; Comolli et al., 2019).

The brachial plexus has been described in several raptors, including *Falco columbarius* (Merlin), *Coragyps atratus* (Black Vulture) , *Buteo buteo* (Common Buzzard), *Rupornis magnirostris* (Roadside Hawk), *Athene cunicularia* (Burrowing Owl), and *Tyto furcata* (American Barn Owl) (Moreira et al., 2009; Cevik-Demirkan, 2014; Silva, 2016; Akbulut et al., 2017; Machado et al., 2021). However, species-specific anatomical and ultrasonographic data remain essential to improve accuracy (Micieli et al., 2021) and reduce iatrogenic injury during regional anesthesia.

Ultrasound-guided techniques have been described in birds, including brachial plexus blocks in Amazona ventralis and Falco tinnunculus, and sacral plexus blocks in *Anas platyrhynchos*, *Gallus gallus*, and *Strix varia* (Cunha et al., 2013; Micieli et al., 2021; Trujanovic et al., 2021; Byrne et al., 2024; Ienello et al., 2024). The axillary approach may reduce the risk of air sac injury compared to dorsal approaches (Debiage, 2025).

The application of ultrasound-guided brachial plexus blocks in birds is limited by the scarcity of species-specific anatomical and ultrasonographic descriptions. To date, no such information is available for *F. sparverius*, despite its frequent presentation for treatment and rehabilitation. Therefore, this study aimed to describe the anatomy and sonoanatomy of the brachial plexus in *F. sparverius*. We hypothesized that the plexus could be consistently identified ultrasonographically, providing an anatomical basis for future regional anesthetic techniques in this species.

## Materials and methods

A total of 19 American kestrels (*F. sparverius*) were included in this study and allocated into three groups according to the research objective: gross anatomical description (n = 6), ultrasonographic evaluation (n = 8), and ultrasound-guided injection with dye dispersion assessment (n = 5). For the anatomical study, six adult individuals admitted for veterinary care at the Wildlife Rehabilitation Center of the Federal University of Pelotas, located in Pelotas, Rio Grande do Sul, Brazil (31°48′03″S, 52°24′30″W), and at the Municipal Zoo of Canoas, located in Canoas, Rio Grande do Sul, Brazil (29°55′00″S, 51°10′07″W), were used. They died or were humanely euthanized from circumstances not related to wing trauma to avoid distortion of the brachial plexus anatomy. Their bodies were immersed in 10% formalin immediately after death and fixed for two weeks. To document the brachial plexus structures, the skin and muscles were carefully dissected bilaterally. The terminology of the Handbook of avian anatomy: Nomina Anatomica Avium (1993) was used (Moreira et al., 2009, Akbulut et al., 2017).

Ultrasonographic evaluation was performed unilaterally in eight live individuals (weighing 85–113 g) under isoflurane anesthesia delivered through a face mask. The specimens were considered clinically healthy and suitable for release. Images were obtained using a portable ultrasonography equipment with a 12-4 MHz linear probe (Lumify Philips®) connected to a 12,4’’ tablet (Samsung® S7) with Lumify software. The specimens were positioned in dorsal recumbency with slight abduction of the wing to the vertebral column and 70% alcohol was used to minimize the interference of feathers (Figure 3). The transducer was placed parallel to the sternum, lateral to the midline, and subsequently advanced in a craniolateral direction over the scapulohumeral joint region. 

For the injection study, five additional cadavers (weighing 91-110 g) were frozen at −20 °C and thawed for 24 hours prior to evaluation. After ultrasonographic localization of the brachial plexus, the needle was inserted using an in-plane approach at approximately 15° relative to the skin surface, progressing from caudal to cranial. Under continuous ultrasound visualization, the needle was advanced craniodorsally through the pectoral musculature until its tip was observed entering the neurovascular sheath surrounding the plexus and subsequently the volume was injected in one spot, using a 22G x 50mm sono-visible needle (Pajunk® SonoPlex, Geisingen, Germany) attached to a 1 mL tuberculin syringe (SR®, Pedro Juan Caballero, Paraguay) primed with 0.5 mL kg-1 of a 3:1 (2% lidocaine:methylene blue - Xylestesin®, Cristalia, Itapira, SP, Brazil: Azul de metileno 1%, Neon Comercial Reagentes Analíticos, Suzano, SP, Brazil- solution (Micieli et al., 2021). Immediately after injection, 0.2 mL of air was injected to flush the needle dead space and ensure delivery of the full injectate volume, which was particularly important given the small volume administered. This injection study was performed unilaterally. Following completion of the injection, the specimens were dissected using the same anatomical approach described for the anatomical study. Dissection was performed immediately after injection to reduce the potential influence of passive dye diffusion. After exposure of the brachial plexus and associated nerve fascicles, dye distribution was evaluated by direct visual inspection.

This study was approved by the Bioethics and Biosecurity Committee of the Federal University of Pelotas (CONCEA-UFPEL; protocol no. 23110.014978/2022-21) and authorized by the Brazilian Biodiversity Information and Authorization System (SISBIO-ICMBio; permit no. 82444-3).

## Results

**Fig. 1:**
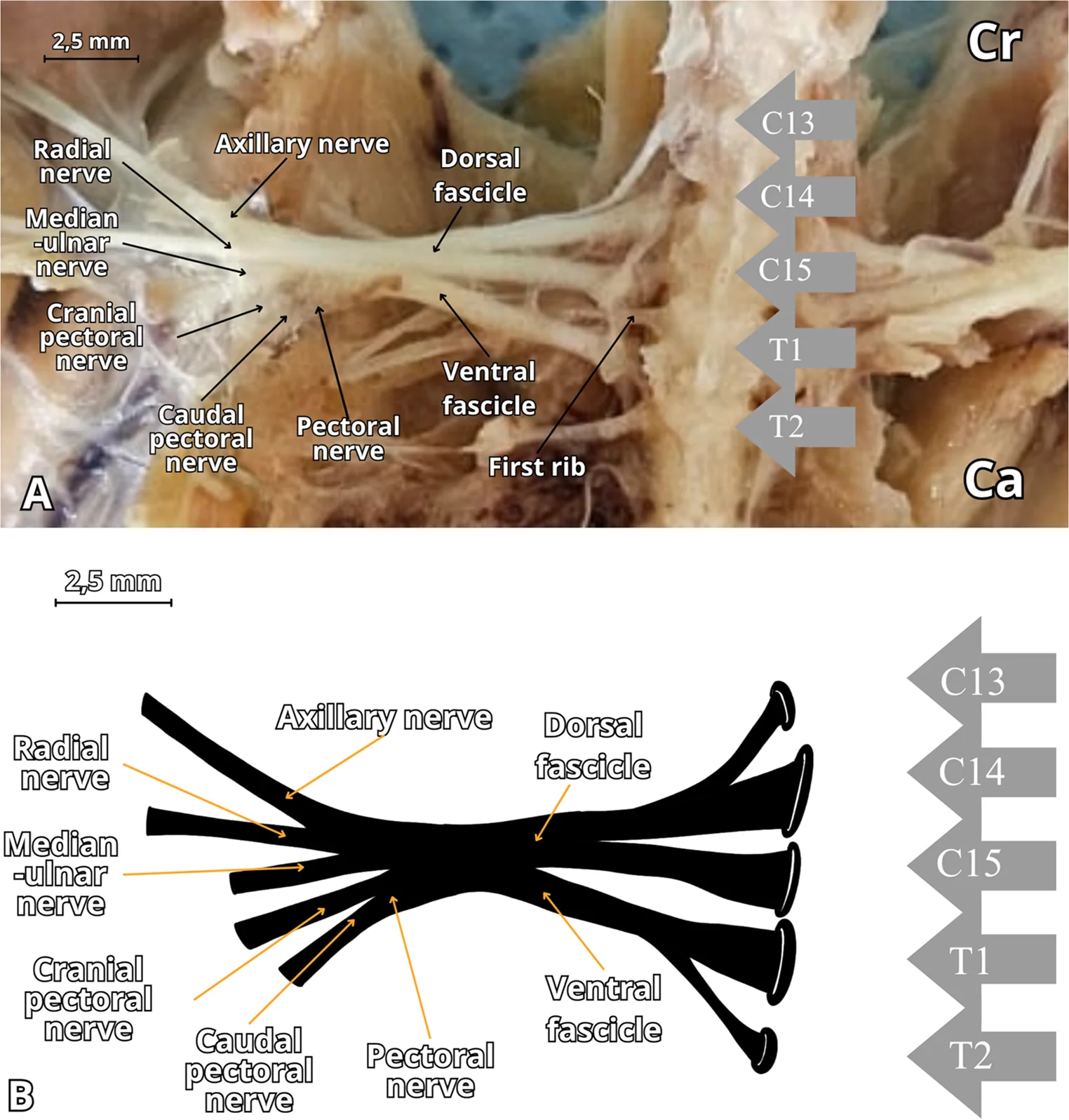
Ventral view of selected nerves comprising the brachial plexus in *Falco sparverius*. Annotated image. (Cr) cranial and (Ca) caudal. Grey arrows indicate the emergence ventral fascicles of the cervical spinal nerve forming the brachial plexus from the intervertebral foramina; first vertebral rib; the dorsal and ventral fascicles of the brachial plexus; axillary nerve (nervus axillaris) radial nerve (nervus radialis); median-ulnar nerve (nervus medianoulnaris); pectoral nerve (nervus pectoralis); cranial pectoral nerve (nervus pectoralis cranialis); caudal pectoral nerve (nervus pectoralis caudalis)

Upon evaluating the anatomy of six *F. sparverius* individuals with a median body weight of 99 g (82–123 g), the presence of 14 cervical vertebrae and six thoracic vertebrae was observed, the brachial plexus was formed by ventral rami of spinal nerves C13 to T2, which emerged bilaterally from the intervertebral foramina as nerve roots (Figure 1). The plexus exhibited an organization with dorsal and ventral fascicles arising from the ventral rami of C13–T2. Once formed, the plexus could be distinguished sequentially into a dorsal fascicle and a ventral fascicle (Figure 1). The dorsal fascicle gave rise to a group of nerves known as the dorsal thoracic nerves and dorsal brachial nerves, responsible for innervating muscles associated with the scapula, flight musculature, and also issuing numerous cranial fascicles that formed a small accessory plexus. The ventral fascicle comprised the ventral thoracic nerves and ventral brachial nerves, which primarily innervated the flight musculature.

The dorsal thoracic nerves innervated both superficial and deep rhomboid muscles as well as the superficial and deep serratus muscles. The ventral thoracic nerves gave rise to the middle thoracic nerves, subscapular nerves, and the pectoral nerve. The dorsal brachial nerves included the axillary, radial, and anconealis nerves, while the ventral brachial nerves encompassed the caudal brachial cutaneous nerve and the median-ulnar nerve.

**Fig. 2:**
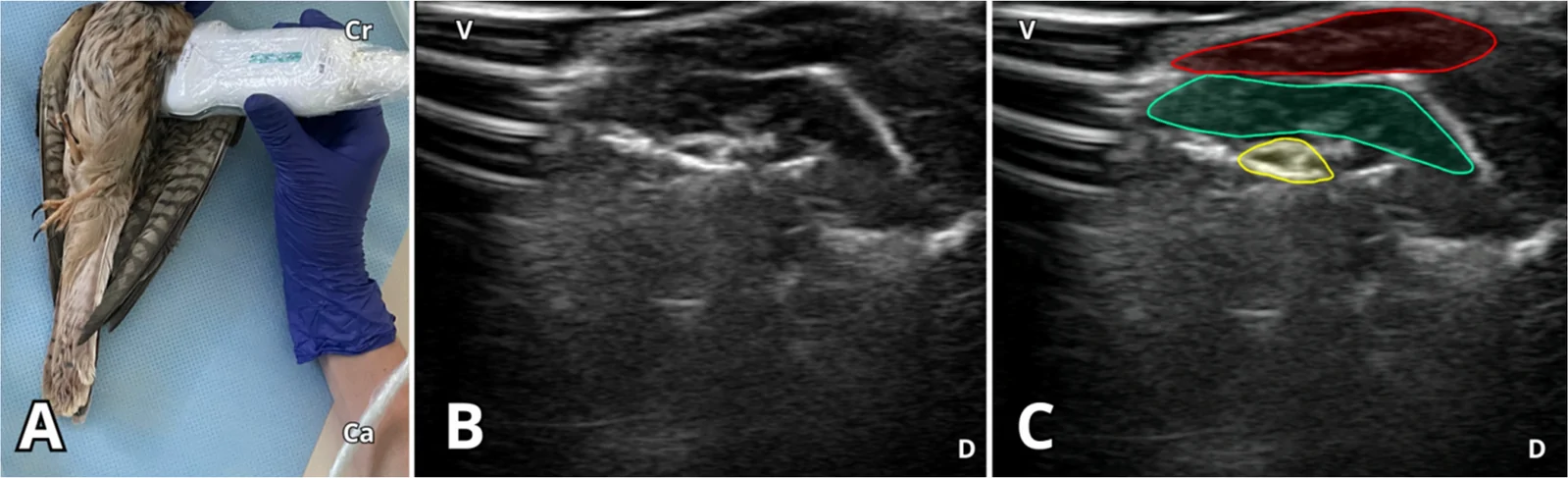
(**A**) Positioning of a 12–4 MHz linear ultrasound probe over the superficial pectoral muscle on the left wing of an American kestrel (*F. sparverius*), focusing on the axillary region (ventral approach) for needle insertion during brachial plexus block. (Cr) cranial and (Ca) caudal. (**B**, **C**) Ultrasonographic anatomy of the brachial plexus using a ventral approach in *F. sparverius*. (**B**) Ultrasound image in a dorsal plane of the brachial plexus. (**C**) Ultrasound image in a dorsal plane of the brachial plexus with schematic anatomical identification. (**D**) dorsal. (**V**) ventral. Red outline: superficial pectoral muscle; green: deep pectoral muscle; yellow: brachial plexus located 0.875 cm from the skin surface

On ultrasonographic examination, the brachial plexus was identified as small hypoechoic structures positioned beneath the superficial and deep pectoral muscles, at a mean distance of 0.875 cm from the skin surface (Figure 2).

**Fig. 3:**
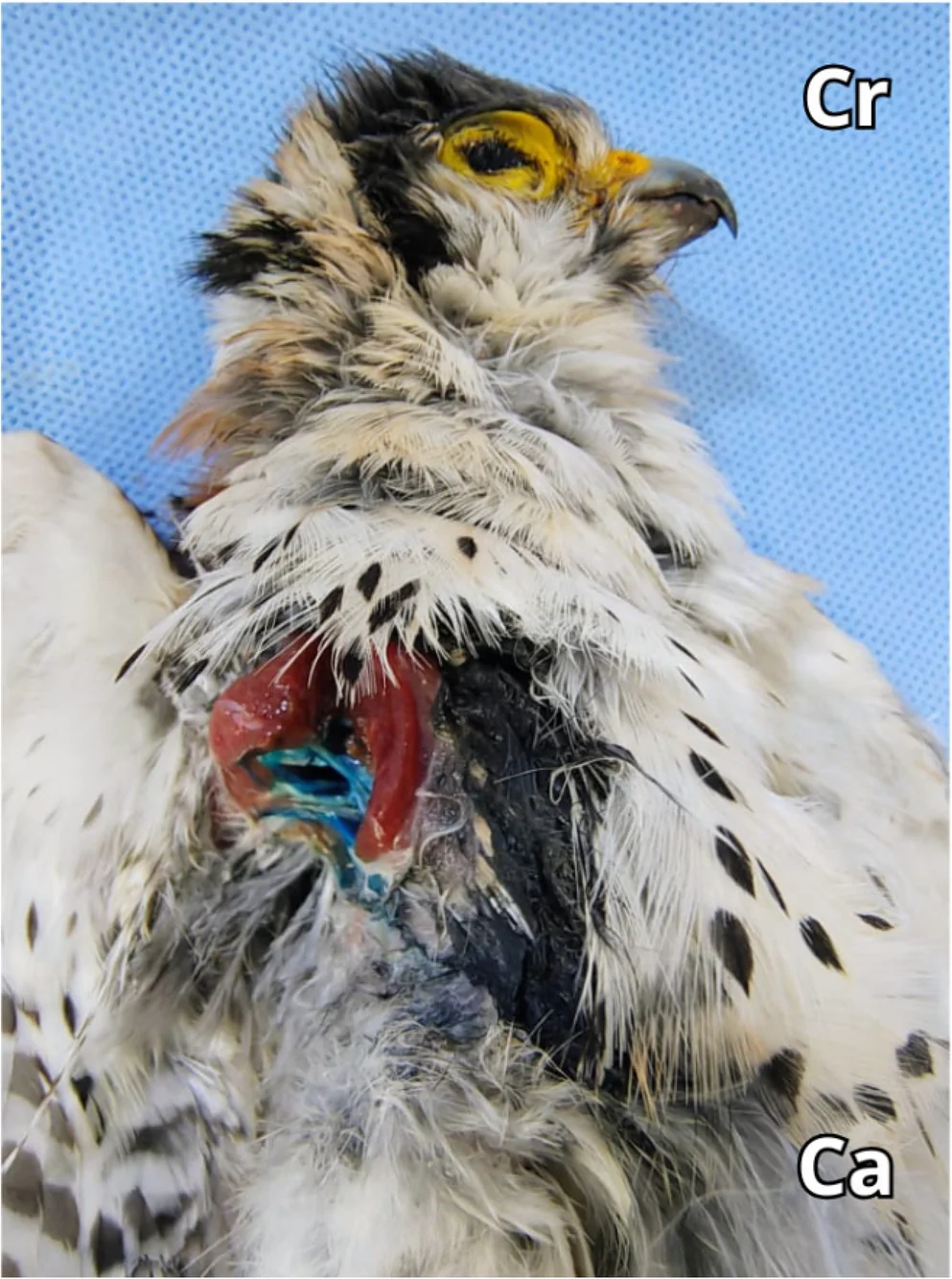
Dissection of the right wing of an American kestrel (*F. sparverius*) cadaver following ultrasound-guided injection of a solution containing 0.5 mL kg-1 of a 3:1 (2% lidocaine:methylene blue, demonstrating dye distribution within the brachial plexus). (Cr) cranial and (Ca) caudal

Ultrasound-guided injection of 0.5 mL kg-1 of a 3:1 2% lidocaine:methylene blue was performed in five *F. sparverius* cadavers using a sono-visible needle. Dye distribution demonstrated consistent anatomical spread over the brachial plexus (Figure 3), with no evidence of vascular puncture or damage to the air sacs.

## Discussion

The brachial plexus of F. sparverius exhibited a typical avian organization, with dorsal and ventral fascicles originating from the ventral rami of C13–T2. The contributions from both cervical and thoracic spinal nerves have also been observed in several other avian species, including the blue-fronted amazon (*Amazona aestiva*) (C9–T2) (Silva et al., 2015), black vulture (*Coragyps atratus*) (C10–T2) (Moreira et al., 2009), common buzzard (*Buteo buteo*) (C11–T2) (Akbulut et al., 2017), and roadside hawk (*Rupornis magnirostris*) (C9–T3) (Machado et al., 2021). In contrast, the exclusive contribution of cervical spinal nerves resembles that reported in the rock pigeon (*Columba livia*) (Franceschi et al., 2009). Despite these differences in segmental origin, the gross anatomical organization of the brachial plexus was conserved, supporting the identification of the plexus by ultrasonography and the application of ultrasound-guided regional anesthesia across avian species. 

In the rock pigeon the brachial plexus includes a well-defined accessory brachial plexus, with contributions from sympathetic nerves and a greater number of fascicles to muscles such as the rhomboideus and serratus (Baumel et al., 1993; Franceschi et al., 2009). In *F. sparverius*, a thin fascicle from the last two cervical sympathetic nerves merged with the plexus formation, indicating a more compact organization, possibly adapted to the smaller body size and flight pattern of this species.

Even in taxonomically closely related species with similar muscular and flight patterns (Mosto et al., 2022), the origin of the radial nerve showed interspecific variation within the brachial plexus. In *F. sparverius*, the radial nerve originates from C13–C14, very similar as in *Gallus gallus domesticus*, it arises from C13–C14 and T1 (Nickel et al., 1977; Dursun, 2002). Whereas in common buzzard (Akbulut et al., 2017) and in the merlin (Cevik-Demirkan, 2014) the radial nerve originates from C12-C13. The median-ulnar nerve, formed by the ventral rami of C14, C15, and T1, showed a pattern similar to that described in domestic birds and ostriches, dividing into the median and ulnar nerves on the medial aspect of the elbow joint (Nickel et al., 1977; Dursun, 2002; Pospieszny et al., 2009) differing from the blue-fronted common buzzard (Akbulut et al., 2017) where it originates from C13, T1 and T2. The pectoral nerve originates from C13 and C14, differing from that described for Falco columbarius, in which it originates from C11 to C12 (Cevik-Demirkan, 2014). This nerve may be absent in ostriches due to pectoral muscle regression (Pospieszny et al., 2009). These observations highlight that, although the overall organization of the brachial plexus is conserved among birds, variations in nerve origin and branching patterns occur between species, which may have implications for the accuracy and reliability of regional anesthetic techniques.

Regarding ultrasonographic anatomy, the brachial plexus of *F. sparverius* was successfully identified using a ventral axillary approach with the ultrasound probe positioned over the superficial pectoral muscle. This approach is similar to that described in *Strix varia* by Byrne et al. (2024), in which successful staining of the brachial plexus was achieved following ultrasound-guided localization. 

In contrast, a dorsal approach has been described for brachial plexus localization in *Falco tinnunculus* (Micieli et al., 2021). Although both approaches allow identification of the plexus, the ventral approach used in the present study avoided advancing the needle toward the clavicular and cervical air sacs, structures that are anatomically prominent in birds and may increase the risk of inadvertent puncture during dorsal approaches (Debiage, 2025). 

Despite the smaller body size of *F. sparverius *compared with *S. varia*, the brachial plexus was consistently visualized in all specimens, demonstrating that ultrasonographic identification is feasible even in small-bodied raptors. These findings suggest that the ventral axillary approach may represent a practical and safe technique for brachial plexus localization in this species. 

Despite the smaller body size of *F. sparverius*, the ventral approach allowed consistent dye distribution within the brachial plexus region following ultrasound-guided injection. These findings support the anatomical feasibility of this approach in this species, even in small-bodied birds.

A total volume of 0.5 mL kg-1 was used in this study, based on previously reported dosing in *F. sparverius* (Micieli et al., 2021). This volume is lower than that reported in similar cadaveric studies, in which larger volumes of diluted local anesthetics have been used, as 1 mL kg-1 of 0.5% bupivacaine (Debiage et al., 2025). Despite the reduced volume, consistent dye distribution was observed in the present study, suggesting that smaller injection volumes may be sufficient to achieve adequate anatomical coverage of the brachial plexus in small avian species. Additionally, in very small birds such as F. sparverius, factors such as needle size and limited anatomical space may influence injectate distribution and should be considered when selecting injection volumes and techniques.

Limitations of this study include the relatively small sample size and the use of cadavers for dye-distribution assessment, which may not completely replicate in vivo conditions. In addition, the clinical efficacy and safety of brachial plexus blockade were not evaluated and should be investigated in future studies. Although consistent anatomical findings were observed among the specimens examined, anatomical variation may exist across individuals, age classes, and geographic populations and could not be assessed within the scope of this study. 

## Conclusion

The origin of the brachial plexus of *Falco sparverius* and its location shows a typical avian pattern with species-specific features, including a more compact structure. Ultrasonographically, it appears as small hypoechoic structures beneath the superficial and deep pectoral muscles via a ventral approach. Ultrasound-guided injection achieved consistent distribution within the plexus region, supporting the anatomical feasibility of this technique.Nerve distribution follows classical avian patterns but with adaptations similar to other falcons (e.g., *Falco columbarius*). Although adequate dye spread was achieved with 0.5 mL kg⁻¹, this study did not assess block efficacy, analgesic success, or procedural safety in live animals. Therefore, the clinical applicability of this technique remains to be determined. Future in vivo studies are required to evaluate the effectiveness, safety, and analgesic outcomes of ultrasound-guided brachial plexus blocks in *F. sparverius*. These findings provide anatomical and ultrasonographic reference data that may support the future development of regional anesthesia techniques in this species. 

## Data Availability

No datasets were generated or analysed during the current study.
